# Status of and Challenges in Therapy of Mucinous Ovarian Cancer Associated with Pseudomyxoma Peritonei Syndrome: Review of Current Options and Future Treatment Trends

**DOI:** 10.3390/life14111390

**Published:** 2024-10-29

**Authors:** George Pariza, Carmen Mavrodin, Alina Potorac, Octavian Munteanu, Monica Mihaela Cirstoiu

**Affiliations:** 1 5th Department of General Surgery, Emergency Hospital Bucharest, 050098 Bucharest, Romania; george.pariza@umfcd.ro; 2 Faculty of Medicine, “Carol Davila” University of Medicine and Pharmacy, 050474 Bucharest, Romania; octav_munteanu@yahoo.com (O.M.); dr_cirstoiumonica@yahoo.com (M.M.C.); 3 3th Department of General Surgery, Emergency Hospital Bucharest, 050098 Bucharest, Romania; 4 Department of Obstetrics and Gynecology, Emergency Hospital Bucharest, 050098 Bucharest, Romania; alinawiki29@gmail.com

**Keywords:** pseudomyxoma peritonei, mucinous ovarian cancer, ovary, ovarian cancer, mucin, oncology, oncologic surgery

## Abstract

Objective: Pseudomyxoma peritonei (PP) is a rare condition, and differentiating between primary and secondary ovarian causes is crucial for determining the appropriate oncological therapy. Given the resistance of ovarian mucinous carcinoma to standard platinum-based chemotherapy, the objective of this review is to present the current therapeutic approaches and summarize the emerging trends in the treatment of this disease. Methods: The authors conducted an exhaustive evaluation of studies published over a 14-year period (June 2010–May 2024) concerning pseudomyxoma peritonei, mucinous ovarian carcinoma, ovarian causes of PP, and ovarian cancer using the following databases: PubMed, Scopus, and Science Direct. The Preferred Reporting Items for Systematic Reviews and Meta-Analyses (PRISMA) guidelines were followed. The results were organized into seven subchapters and analyzed. Results: The analyzed studies present surgery followed by HIPEC as the current therapy with the best long-term survival results. However, the oncological treatment is unsatisfactory, and the choice of therapy depending on the primary origin of the tumor becomes particularly important. For the differential diagnosis between pseudomyxoma due to a gastrointestinal cause and that of ovarian origin, genetic analyses are recommended; these include the characteristics of the mucin present in the lesion, as the therapeutic response can have contradictory results depending on the primary origin of the tumor. Conclusions: Surgery followed by HIPEC remains the standard for resectable cases. However, oncological treatment has controversial results in the case of mucinous ovarian carcinoma compared to other types of ovarian cancer and to metastatic ovarian tumors associated with pseudomyxoma of the peritoneum. Based on the articles included in this review, it was found that the current trend is the study of mucin as a resistance factor against chemotherapy based on platinum products and the targeting of oncological therapy according to the tumor’s genetic characteristics.

## 1. Introduction

Pseudomyxoma peritonei (PP) is a rare clinical syndrome, with an incidence of 1–2 cases per million per year, characterized by the presence of diffuse gelatinous ascites distributed throughout the peritoneal cavity [1,2,3].

The accumulation of intraperitoneal mucin secondary to a mucinous neoplasm with diverse origins and frequencies, along with metastatic implants on the peritoneal surface, creates the clinical picture of PP [1,2,3,4].

The syndrome was first described in 1884 by Werth [2], who introduced the term PP and initially attributed it to perforated appendiceal cystadenomas. Currently, multiple causes have been identified, the most common being appendiceal in origin, followed by digestive causes (small and large intestine, stomach, pancreas, lung, breast, gallbladder, fallopian tubes, and ovaries). An appendiceal origin accounts for 90% of cases, while the remainder are due to rarer causes [1,2,3].

The primary or secondary ovarian causes associated with pseudomyxoma peritonei are listed below [4,5]:

- Mucinous cystadenoma;
- Mucinous ovarian cancer;
- Ovarian metastasis of gastrointestinal cancer;
- Malignant transformation of an ovarian primary mature cystic teratoma;
- Appendiceal mucocele with carcinomatosis, mimicking an ovarian mucinous carcinoma;
- Mucinous borderline tumor developed within an ovarian teratoma.

Mucinous ovarian carcinoma is a rare genital tumor, with an incidence of approximately 3–5% of epithelial ovarian cancers [1,5].

Most commonly evolving asymptomatically, pseudomyxoma peritonei is often discovered incidentally at a relatively advanced stage of the disease during laparoscopy, laparotomy, or imaging investigations for other medical conditions [1,4]. Pseudomyxoma peritonei can be considered a borderline malignant disease, with prognosis closely correlated with pathological classification (Peritoneal Surface Oncology Group—PSOG) [4,6].

Although the standard treatment—surgical cytoreduction combined with hyperthermic intraperitoneal chemotherapy (HIPEC)—has improved the prognosis of these patients, issues related to intraoperative difficulties, tumor recurrence, and poor quality of life remain unresolved, leaving room for further biological research into understanding this pathology, as well as efforts to identify alternative therapies [2,7].

## 2. Materials and Methods

The authors conducted an exhaustive evaluation of studies published over a 14-year period (June 2010–May 2024) concerning pseudomyxoma peritonei, mucinous ovarian carcinoma, ovarian causes of PP, ovarian cancer, and the ovaries using the following databases: PubMed, Scopus, and Science Direct. The results were organized into 7 subchapters and analyzed. This research study had the following selection criteria: articles in English with the subject of medicine, and research conducted on humans. Articles in English were selected, and case presentations and case series were excluded.

### 2.1. Natural History and Characteristics of PP with Ovarian Origin

The estimated incidence of pseudomyxoma peritonei (PP) is very rare, approximately 1 to 4 cases per million diagnosed annually. The most frequently identified primary sites are mucinous appendiceal or ovarian adenocarcinomas. Patients have a median age of 53 years at diagnosis, with women being more commonly affected than men [1,2,4,6].

PP occurs secondary to mucin production by peritoneal metastatic implants arising from the perforation of a mucinous neoplasm. The rupture of the primary tumor leads to the release of mucin and epithelial cells, which subsequently implant on the peritoneum. The implantation process is influenced by gravity and areas of fluid absorption. Major absorption areas include the greater and lesser omentum and diaphragmatic surfaces, particularly the right diaphragm, leading to diaphragmatic accumulations and tumoral transformation of the omentum. The second migration mechanism, gravity, causes the accumulation of neoplastic cells in the rectovesical space, retrohepatic space, and paracolic gutters [2,4,7]. Mobile viscera, such as the small intestine, are spared in the early stages of the disease, allowing for R0 tumor resection in initial phases without extensive intestinal resection. Tumor localization on the intestinal serosa and the mesenteric–enteric junction makes complete excision difficult and is a predictor of inoperability [2,7,8].

Once implanted, the tumor cells continue to proliferate and produce a large amount of mucus, forming mucinous ascites over a period of months or even years. Progressive complications of PP, such as intestinal obstruction, may necessitate urgent surgical intervention. Cases of mucinous invasion into the pleural cavity have been reported. However, it should be noted that lymphatic or hematogenous metastasis in PP is rare [2,4,6,7,8].

### 2.2. Clinical and Imaging Evaluation

Diagnosing PP remains a challenge for clinicians, with 50% of cases being asymptomatic or incidentally diagnosed [9,10]. In rare instances, patients may present with abdominal pain, weight loss, nausea, or vomiting; they may also present with palpable intra-abdominal masses, mimic acute appendicitis, or display various gynecological conditions. In women, infertility may be one of the manifestations when the disease involves the ovary or pelvis. More advanced disease can lead to abdominal distension, ascites, bowel obstruction, and nutritional impairment [2,7,9].

**Figure 1:**
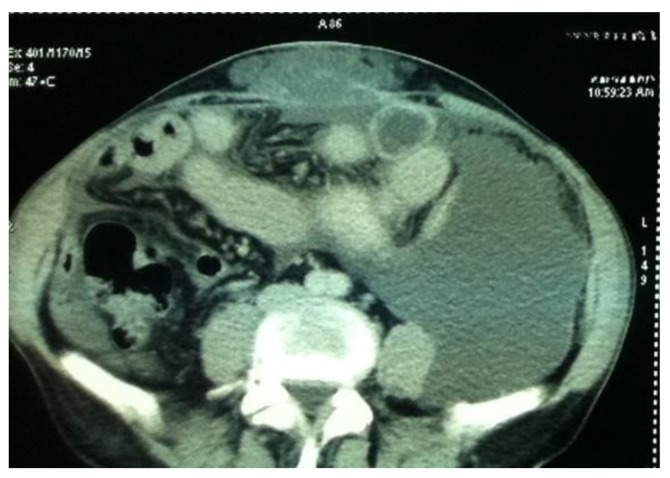
Pseudomyxoma of the peritoneum with subcutaneous mucin extravasation; clinical differential diagnosis with an incarcerated incisional hernia.

Imaging is essential in differential diagnosis and the diagnosis of the primary tumor, as well as in the assessment of the index of peritoneal carcinomatosis and tumor recurrence (Figure 1).

Currently, contrast-enhanced abdominopelvic computer tomography (CT) is the imaging modality of choice for diagnosing pseudomyxoma peritonei [9,10,11,12]. The typical CT appearance of the liver and spleen surfaces is scalloped, caused by loculated mucin accumulations, which helps distinguish mucin from fluid ascites [10,11].

**Figure 2:**
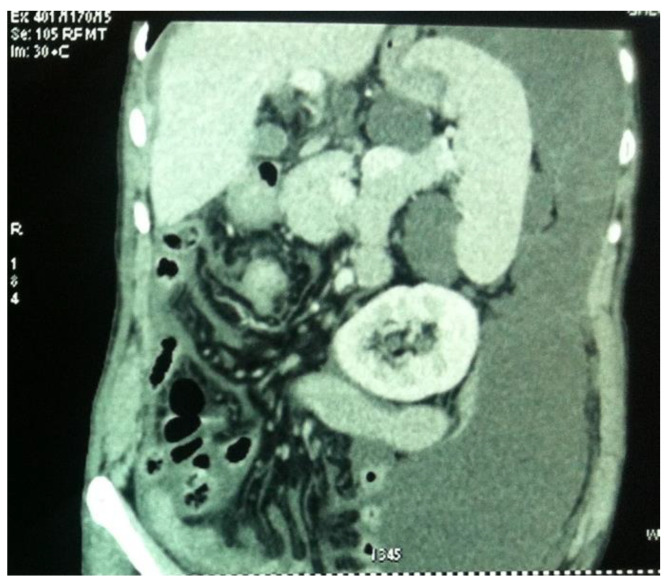
CT aspect of PP; evaluation of secondary peritoneal and intestinal metastases is affected by the presence of mucin in the left flank.

However, CT has a limited performance in the detection of metastatic adenopathy, with a sensitivity of 41% and a specificity of 89%, a fact that influences the decision regarding infrarenal lymphadenectomy [13]. In addition to underestimating metastatic adenopathy, CT also underestimates the presence of peritoneal metastases, especially in the presence of ascites or mucin accumulations specific to pseudomyxoma (Figure 2).

Therefore, the peritoneal carcinomatosis index PCI) is underestimated in CT evaluations. This influences the decision regarding the therapeutic attitude and the selection of patients for cytoreduction or neoadjuvant chemotherapy (Table 1).

However, the primary lesion may be absent or difficult to identify on imaging. Compared to CT, MRI is more sensitive in locating the tumor and evaluating the small intestine and hepatoduodenal ligament, as well as in differentiating fluid ascites from gelatinous ascites [10,11,12].

An advantage of MRI, compared to CT, is represented by its ability to detect small peritoneal metastases, a fact that places MRI in the first line in the evaluation of candidate patients for cytoreduction [12,13].

PET/CT scanning may be useful in more aggressive variants by detecting extra-abdominal spread, as well as in the preoperative assessment of PP’s pathological grade and the feasibility of achieving complete cytoreduction [11,12].

PET CT is not recommended, due to low specificity, in the initial diagnosis of the primary tumor, especially in tumor types with a clear-cell and mucinous-invasive subtype. However, PET CT is superior to CT in the determination of metastatic lymph nodules, secondary peritoneal determinations, and recurrences [13].

### 2.3. Pathology

From a macroscopic standpoint, pseudomyxoma peritonei (PP) presents with mucinous ascites accompanied by cystic epithelial implants on peritoneal surfaces, with lesions varying in size from a few millimeters to several centimeters [14].

Several histological grading systems have been proposed for PP, and they are highly indicative of the disease’s prognosis. The World Health Organization (WHO) refined the histological classification system for PP based on the studies by Ronnett et al. [4,7]. In 1995, Ronnett’s team divided this pathology into two categories: disseminated peritoneal adenomucinosis (DPAM) and peritoneal mucinous carcinomatosis (PMCA). DPAM is characterized by abundant mucin containing few mucinous epithelial cells, with minimal cytological atypia and low mitotic activity, while PMCA is characterized by more abundant mucinous epithelial cells with significant cytological atypia and high-grade mitotic activity [4,7,14].

In 2010, the American Joint Committee on Cancer (AJCC) and WHO proposed a histological classification of PP based on the understanding of histogenesis, molecular genetic findings, and the clinical behavior of these lesions [2,7,15]. This classification divides PP into the following types:

1. **Acellular mucin**—mucin within the peritoneal cavity without neoplastic epithelial cells.
2. **Low-grade mucinous carcinoma peritonei** (synonymous with **DPAM**)—mucin with low cellularity (less than 10%) and without infiltrative growth.
3. **High-grade mucinous carcinoma peritonei** (synonymous with **PMCA**)—mucin with high cellularity, moderate/severe cytological atypia, numerous mitoses, and a cribriform growth pattern. Infiltrative invasion of the underlying organs is often present.
4. **High-grade mucinous carcinoma peritonei with signet ring cell** is classified separately because of its worse prognosis.

### 2.4. Differential Diagnosis of Secondary and Primary Ovarian Tumors and Pathological Subtypes

Statistically, 65–80% of mucinous ovarian carcinomas (MOCs) are diagnosed at early stages [16]. Patients diagnosed with MOC in FIGO Stage I have a 5-year survival rate of nearly 90% [16,17,18]. However, survival rates in FIGO Stages II-IV are poorer compared to those diagnosed with serous ovarian cancer at similar stages. The average survival for patients with MOC diagnosed in Stages III-IV is between 12 and 33 months [17,18,19].

Identifying pathological subtypes and differentiating primary ovarian tumors from secondary ones poses significant challenges. While the prognosis is favorable in early stages, advanced cases have poor survival, which can also be attributed to the tumor’s resistance or suboptimal response to chemotherapy [17,18,19]. Given that gastrointestinal mucinous tumors and MOC share similar molecular characteristics, retrospective studies have shown that patients with MOC have responded to empirical therapies with chemotherapy specific to gastrointestinal tumors [19,20].

The exact cause of the poor response to platinum-based standard therapies used in ovarian neoplasms is unclear, and prospective studies are difficult to conduct due to the rarity of this pathology [16,21]. Therefore, recent efforts to identify new therapeutic strategies have focused on analyzing the pathological and molecular aspects of MOC [16,21,22].

Pathological evaluation of mucinous ovarian tumors has shown that the vast majority of these are secondary, with origins most commonly in the gastrointestinal tract, breast, cervix, and others, while primary mucinous ovarian tumors have a frequency of only 1–3%. Identifying the primary origin is crucial for choosing the appropriate therapy and establishing prognosis [21,22].

Standard methods for the differential diagnosis between primary (PMOCs) and secondary ovarian mucinous tumors (MMOCs) include clinical aspects, pathological anatomy, and immunohistochemistry [16]. Primary tumors typically present as large ovarian masses, often greater than 10 cm, with unilateral ovarian lesions, a normal-looking appendix on imaging or intraoperatively, absence of serosal or capsular tumor implants, absence of primary gastrointestinal lesions, and absence of extracellular mucin [2]. The presence of benign or borderline tumor components supports a primary ovarian origin of mucinous peritonitis. Secondary lesions often involve the ovarian hilum and surface, show infiltrative stromal invasion, extracellular mucin synthesis, and widespread distribution of signet ring cells [2,5].

Frequently used immunohistochemical markers include CK7, CK20, CDX2, PAX8, estrogen, and progesterone [23,24,25]. Recently, new markers have been introduced, whose combined analysis may improve the differential diagnosis between PMOC and MMOC. The combination of SATB2 and CK7 has high accuracy in differentiating gastrointestinal mucinous tumors from primary ovarian mucinous tumors [23,24,25,26].

According to the NCCN, PAX8 immunostaining is typical of primary ovarian tumors, although the absence of PAX8 does not rule out the ovary as the primary site, while SATB2 is consistent with colonic origin. Metastatic colorectal adenocarcinomas also typically express CK20 and CEA [24,25].

The microscopic pattern of mucin distribution can be used in the differential diagnosis of primary or secondary ovarian origin. Primary tumors have a high level of intracellular mucin and lower levels of extracellular localization, while metastatic tumors exhibit high extracellular expression [16,27].

**Figure 3:**
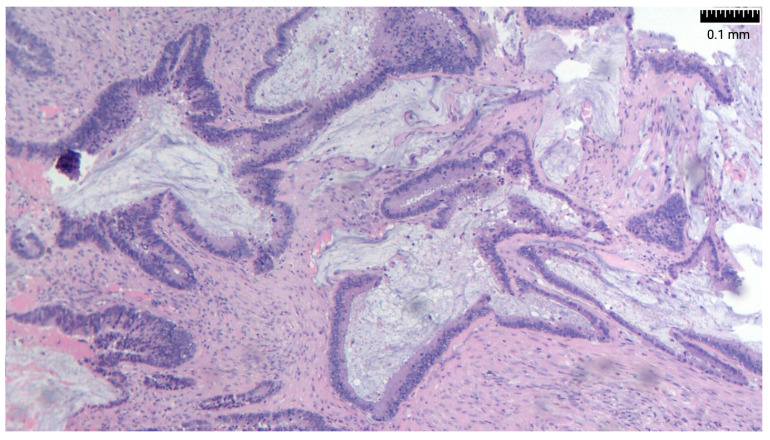
MLH-1: positive nuclear staining in tumor cell.

**Figure 4:**
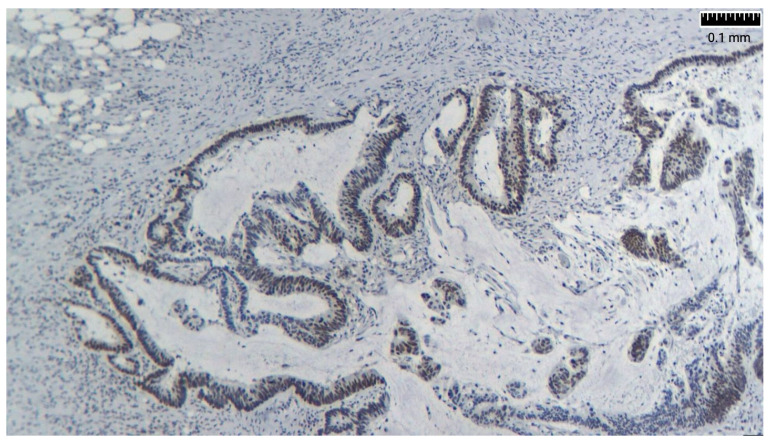
PMS-2: positive nuclear staining in tumor cell.

Researchers have suggested the overexpression of MUC2 as a molecular marker for PP of intestinal origin. Immunohistochemically, appendiceal tumors also express CK20, CEA, and CDX2 and are usually negative for CK7 and CA 125. There are also reports of loss of expression of the mismatch repair genes *MLH-1* and *PMS-2* (Figure 3 and Figure 4) [26,27] (Table 2).

In recent years, research attention has been directed towards the genetic analysis of mucin. In a review published in 2023, Yicong Wang et al. discussed mucin as a factor in chemotherapy resistance and the importance of its genetic analysis for differentiating primary ovarian from secondary tumors, with an impact on chemotherapy treatment selection [16].

An immunohistochemical study on 175 cases, published in 2011 by Chu et al., evaluated the mucin-specific genes *MUC1*, *MUC2*, and *MUC6* in tumors from different locations [16,28]. The study found that only *MUC2* and *MUC6* are useful in determining the primary origin of the tumor. Another retrospective study that analyzed ovarian metastases from colorectal cancer and primary ovarian tumors found *MUC2* gene positivity in 51% of colorectal cancers and 0% in ovarian cancers, while the *MUC5AC* gene was present in 2.4% of metastatic gastrointestinal tumors and 50% of primary ovarian tumors [29].

The differential diagnosis of primary ovarian tumors from metastatic ones involves analyzing a combination of factors, including MUC genes, *PAX8*, *CK20*, and *SATB2*, which can increase diagnostic sensitivity and accuracy, with implications for therapeutic strategies [27,28,29].

### 2.5. Tumor Markers

It has been found that tumor markers have prognostic value in pseudomyxoma peritonei (PP), are useful for monitoring patients after treatment, and may suggest the primary or secondary origin of the ovarian tumor. CA 125 provides the highest diagnostic performance in mucinous ovarian carcinoma, followed by CA 19-9 and CEA.

CEA and CA 19-9 are elevated in PP of gastrointestinal origin, while CA 125 is elevated in conditions involving ovarian pathology [1,6]. Patients with elevated preoperative CEA and CA 19-9 levels have a lower median survival compared to those with negative or low markers [6] (Table 2).

In a recent study published in 2019, which included 225 patients with pseudomyxoma who underwent cytoreductive surgery and HIPEC (mytomycin C, 35 mg/m^2^, at 40–41 °C for 90 min), CEA and CA 19-9 were routinely determined preoperatively in all patients, while CA 125 was inconsistently measured. The follow-up of patients was conducted over a 10-year period or until death and consisted of tumor marker measurements and CT scans. It was observed that patients with acellular mucin had elevated CEA levels in only 11% of cases, while patients with DPAM (disseminated peritoneal adenomucinosis) showed elevated serum levels in 65.8% of cases and 63% in patients with PMCA (peritoneal mucinous carcinomatosis). Overall survival and disease-free survival were significantly lower in patients with DPAM or PMCA histology who underwent CRS and HIPEC. In conclusion, the Dutch study defined CEA and CA 19-9 markers as independent prognostic factors for overall survival in PP patients with gastrointestinal origin [7,30].

### 2.6. Therapeutic Management

Patients diagnosed with mucinous ovarian carcinoma require surgical intervention [2,7]. Considering the risk of metastasis during surgery due to the rupture of cystic tumors, the dissemination of intraperitoneal contents, and their seeding, which can lead to long-term recurrences and worsen the prognosis, intraoperative rupture of the cyst must be avoided at all costs [2,7,16].

Traditional periodic surgical interventions aimed at abdominal decompression are now considered outdated due to the inevitable recurrence of the disease [7]. The currently recommended standard treatment for PP consists of complete cytoreductive surgery (CCRS) followed by hyperthermic intraperitoneal chemotherapy (HIPEC) [2,7].

The surgical principles of cytoreduction in PP overlap with those applicable to peritoneal carcinomatosis secondary to ovarian tumors (Table 3).

Complete cytoreductive surgery (CCRS) is evaluated at the end of the procedure by measuring and noting the diameters of residual tumor deposits (Table 4).

As for the completeness of cytoreduction (CC), CC0 and CC1 are considered CCRS in PP surgery.

HIPEC aims to eradicate any remaining macroscopic or microscopic tumor residues, with mitomycin C (MMC) being the most frequently used chemotherapeutic agent, along with 5-fluorouracil, cisplatin, and oxaliplatin. In cases where CCRS is not possible, maximal tumor debulking can extend survival and improve quality of life [2,30,33].

Medical comorbidities should be carefully evaluated preoperatively, and patients with high-grade lesions or ring-like cell components with an ECOG (Eastern Cooperative Oncology Group) performance score of 2–3 have significantly poorer overall survival after surgery. Such patients may benefit from major palliative resection to improve quality of life [30,32,33].

For advanced patients with extensive tumor dissemination and low possibility of achieving complete cytoreduction, interval debulking surgery after neoadjuvant chemotherapy (NACT) has increasingly been offered as a valid alternative, although several European/Asian randomized controlled trials have demonstrated non-inferior prognosis of NACT against primary debulking surgery [34].

Postoperative systemic chemotherapy has been shown to benefit patients with high-grade neoplasms after CRS/HIPEC, but not those with low-grade neoplasms [2]. Current studies are focusing on understanding the underlying molecular mechanisms involved in this pathology, which may facilitate the identification of personalized therapies for PP patients. *KRAS* and *GNAS* gene mutations are frequently involved in the development of this condition. KRAS mutations have been present in 58–94% of cases and are found in high-grade PP, while GNAS mutations are common in low-grade PP. Conversely, *TP53* and *PI3K-AKT* gene mutations play an essential role in disease progression. [1,7]

According to NCCN recommendations, for stage Ic, 5-FU, leucovorin, oxaliplatin/capecitabine, and oxaliplatin/paclitaxel and carboplatin are currently used, and for stages II-IV, bevacizumab is added to these regimens. However, the survival of patients with advanced stages (FIGO III–IV) of mucinous ovarian carcinoma is inferior to those with serous carcinoma. Mucin is associated with reduced chemotherapy efficacy and is considered in recent studies as a potential therapeutic target [16]. The choice of mucin as a therapeutic target has been analyzed in a series of clinical studies/trials, where mucoprotein was targeted in various types of ovarian cancers. Y. Wang et al. reported that Gatipotuzumab showed good results in clinical trials targeting advanced ovarian cancer but was not effective as maintenance therapy in recurrent ovarian cancer (Table 5).

### 2.7. Prognostic

The prognosis of pseudomyxoma peritonei is closely correlated with histopathological classification. The ten-year survival rate for patients undergoing CRS/HIPEC treatment is as follows: 63% for those with low-grade tumors/DPAM, 40.1% for patients with high-grade tumors/PMCA, and 0% for patients with high-grade tumors with ring-like cells [38].

## 3. Discussion

Before the introduction of CRS and HIPEC as therapeutic standards, the results were unsatisfactory, with surgery consisting of repeated debulking procedures, leading to an approximate 10-year survival rate of 32% for patients with DPAM and a 5-year survival rate of 6% for those with PMCA [39].

An expert consensus in 2008 stated that CRS and HIPEC provide significant survival benefits for patients with PP. In 2012, Chua et al. published the results of a study conducted across 16 centers, including 2,298 patients, showing that the average survival at 3, 5, 10, and 15 years reached 80%, 74%, 63%, and 59%, respectively. The mortality rate was 2%, and the rate of major complications was 24%. For patients with CC0 and CC1, the 5-year survival rate was 85%. These data emphasize the importance of cytoreduction as a prognostic factor. Even in advanced cases where R0 resection is not possible, maximal tumor debulking offers survival benefits. The surgeon must pay close attention to excision to avoid iatrogenic peritoneal contamination with mucus during the resection of mucinous tumors.

Cytoreductive surgeries are associated with a mortality rate of 0.6–44% and morbidity rates ranging from 7% to 49%. Special attention should be given to cases where CC0 or CC1 is achieved, with early recurrence identification being essential. This is achieved through annual imaging (CT) and tumor marker monitoring for 10 years.

The personalization of oncologic therapy depends on identifying the primary origin of mucinous tumors. Genes such as *MUC2*, *MUC5AC*, *MUC6*, and *MUC13*, as well as the predominant intracellular presence of mucin, appear to be involved in the development and recurrence of mucinous ovarian carcinoma and can be used to differentiate between primary ovarian and metastatic lesions. There is a correlation between mucin secretion by tumor cells and chemoresistance; according to Wang et al., the *MUC1* and *MUC6* genes could be considered therapeutic targets. SATB2 is a highly specific marker for gastrointestinal tumors, with no expression in mucinous ovarian carcinoma, making it useful in differential diagnosis.

The resistance of mucinous ovarian tumors to platinum-based chemotherapeutic agents has led to the study of monoclonal antibodies such as oregovomab and abagovomab, which showed initially favorable results but with limited long-term remission effects (Table 5).

Differentiating metastatic ovarian tumors of gastrointestinal origin from primary ovarian tumors is essential in selecting oncologic therapy, especially considering the resistance of mucinous ovarian carcinoma to the platinum-based agents routinely used in ovarian cancer treatment. The challenge in establishing therapeutic protocols lies in the rarity of mucinous ovarian carcinoma, making extensive research on the most suitable therapeutic algorithms difficult.

## Figures and Tables

**Table 1:** ESGO 2017 recommendations for contraindications for CRS (cytoreductive surgery) [13].

- Diffuse deep infiltration of the root of small bowel mesentery	
- Diffuse carcinomatosis of the small bowel involving such large parts	
that resection would lead to short bowel syndrome (remaining bowel < 1.5 m)	
	- Diffuse involvement/deep infiltration of the following:
	- Stomach/duodenum;
	- Head or middle part of the pancreas;
	- Involvement of coeliac trunk, hepatic arteries, left gastric artery;
	- Central or multisegmental parenchymal liver metastases;
	- Multiple parenchymal lung metastases (preferably histologically proven).
- Non-resectable lymph nodes	
- Brain metastases	

**Table 2:** Characteristics of primary and secondary tumors in PP.

Tumor Origin	Mucin Characteristics	Tumor Markers	IHC Markers	
Primary ovarian tumor	High level of intracellular mucin	Elevated CA 125	PAX8 (typical for primary ovarian but inconsistent)	
Secondary (metastatic gastrointestinal) ovarian tumor	High level of extracellular mucin (acellular mucin)	Elevated CA 19-9 CEA	SATB2 (consistent)	*MUC2* CK20

**Table 3:** NCCN Guidelines, Version 3.2024. Principles of surgery [30,31].

*Mandatory Objectives of Cytoreductive Surgery in PP*
As with a primary debulking procedure, every effort should be made to achieve maximum cytoreduction during an interval debulking procedure. Maximal effort should be made to remove all gross disease in the abdomen, pelvis, and retroperitoneum.
All peritoneal surfaces should be visualized, and any peritoneal surface or adhesion suspicious for harboring metastasis should be selectively excised or biopsied.
A radical greater omentectomy should be performed.
Procedures that may be considered for optimal surgical debulking include bowel resection and/or appendectomy, stripping of the diaphragm or other peritoneal surfaces, splenectomy, partial cystectomy and/or ureteroneocystostomy, partial hepatectomy, partial gastrectomy, cholecystectomy, and/or distal pancreatectomy.
In primary invasive mucinous tumors of the ovary, the upper and lower GI tract should be carefully evaluated to rule out an occult GI primary with ovarian metastases, and an appendectomy need only be performed in patients with a suspected or confirmed mucinous ovarian neoplasm if it appears to be abnormal. A normal appendix does not require surgical resection in this setting. If a mucinous histology is confirmed by intraoperative frozen section analysis and there are no suspicious lymph nodes, omitting lymphadenectomy should be considered. *(after National Comprehensive Cancer Network Gudelines 2024.)*

**Table 4:** Completeness of cytoreduction (CC) [2,32,33,34].

CC0	complete removal of all visible disease
CC1	residual disease less than 0.25 cm
CC2	residual tumor deposits between 0.25 and 2.5 cm
CC3	residual tumor deposits > 2.5 cm

**Table 5:** Mucin-targeted therapies in clinical trials (after Wang et al.) [16].

Authors	Year	Therapeutic Agents	Study	Types of Cancers Eligible	Total Patient	Pathological Types	Findings
(Liu et al., 2021) [35]	2021	DMUC4064A (ADC-MUC16)	Phase I	Treatment in patients with platinum-resistant recurrent ovarian cancer	65	N/R	The clinical benefit rate was 42%. 27 patients had CR, or PR or SD lasting ≥ 6 months
(Brewer et al., 2020) [36]	2020	Oregovomab (MUC16)	Phase II	Front-line chemoimmunotherapy with carboplatin-paclitaxel using oregovomab indirect immunization in advanced ovarian cancer	97	Mucinous—2 Serous—86 Endometrioid—6 Clear cell—2 Other—1	Significant improvement in PFS and OS. Prolonged PFS 29.6 months for the oregovomab group. OS has not yet been reached.
(Ledermann et al., 2022) [37]	2022	Gatipotuzumab (MUC1)	Phase II	Maintenance therapy of patients with recurrent epithelial primary ovarian, fallopian tube, or primary peritoneal cancer	216	N/R	No improvement in PFS or OS observed.

N/R—not reported; CR—complete response; PR—partial response; PFS—progresion-free survival; OS—overall survival.
